# New challenges for text mining: mapping between text and manually curated pathways

**DOI:** 10.1186/1471-2105-9-S3-S5

**Published:** 2008-04-11

**Authors:** Kanae Oda, Jin-Dong Kim, Tomoko Ohta, Daisuke Okanohara, Takuya Matsuzaki, Yuka Tateisi, Jun'ichi Tsujii

**Affiliations:** 1 Department of Computer Science, Graduate School of Information Science and Technology, University of Tokyo, 7-3-1 Hongo, Bunkyo-ku, Tokyo, Japan; 2 Faculty of Informatics, Kogakuin University, 1-24-2 Nishi-shinjuku, Shinjuku-ku, Tokyo, Japan; 3 School of Computer Science, University of Manchester, Oxford Road, Manchester, M13 9PL, UK; 4 National Centre for Text Mining, 131 Princess Street, Manchester, M1 7DN, UK

## Abstract

**Background:**

Associating literature with pathways poses new challenges to the Text Mining (TM) community. There are three main challenges to this task: (1) the identification of the mapping position of a specific entity or reaction in a given pathway, (2) the recognition of the causal relationships among multiple reactions, and (3) the formulation and implementation of required inferences based on biological domain knowledge.

**Results:**

To address these challenges, we constructed new resources to link the text with a model pathway; they are: the GENIA pathway corpus with event annotation and NF-kB pathway. Through their detailed analysis, we address the untapped resource, ‘bio-inference,’ as well as the differences between text and pathway representation. Here, we show the precise comparisons of their representations and the nine classes of ‘bio-inference’ schemes observed in the pathway corpus.

**Conclusions:**

We believe that the creation of such rich resources and their detailed analysis is the significant first step for accelerating the research of the automatic construction of pathway from text.

## Background

Originally created as a graphical depiction of biological knowledge, the pathway has developed into a way of organizing biological knowledge [1,2]. Pathways are becoming increasingly important for bio-medical research, since they represent collectively attested interpretations of a large number of facts scattered throughout literature. As such, Text Mining (TM) tools that facilitate the construction and maintenance of pathway knowledge bases have become indispensable tools for biologists to manage the ever-increasing quantity of biological literature.

A few TM systems have been developed for automatic bio-network construction by extracting binary interactions between proteins or genes [3-6]. While the resultant networks appear to be pathways, they do not represent any coherent interpretations of the reported facts. To transform the results of automatically constructed networks to pathways seems to require further efforts which emulate the interpretations of biologists, including inferences based on biological background knowledge.

In this study, we took on a very different approach from previous works. We first examined how a biologist would construct a pathway from a given set of articles by recording which sentences in the articles the biologist found useful for the construction of the portions of a pathway. Then, we formulated what difficulties TM techniques should resolve in order to facilitate a manual curation process for pathways. The results show that pathway construction involves much more challenging tasks for the current TM technology than we had initially assumed.

The main challenges are classified into three groups: (1) the identification of the mapping position of a specific entity or reaction in a given pathway, (2) the recognition of the causal relationships among multiple reactions, and (3) the formulation and implementation of required inferences based on biological domain knowledge. Inferences that biologists make to associate information in text with pathway (concrete representation of interpretation) seem quite different from deduction. Certain expressions in text trigger inferences, which are abductive in nature, to relate them with specific interpretations. Though this type of inference is pervasive in the process of understanding text and unspecific to the biology domain, we call them “bio-inferences” for the sake of brevity.

In this paper, we report a detailed corpus study, which leads to the formulation of these three challenges. We expect the results will contribute to the design of an intelligent TM tool kit for pathway construction and maintenance. The study has been conducted by adding new annotations to a subset of the GENIA corpus [7,8], which associates sentences with corresponding portions in a pathway, and compares them with event annotations independently made to the GENIA corpus.

## Results

### PPI network and manually curated pathways

To demonstrate the differences between manually constructed pathways and PPI networks, we take the Toll-like receptor (TLR) pathway as a typical example of a manually constructed pathway [9]. The pathway, which was constructed based on 411 publications and is one of the largest of its kind, consists of 652 nodes and 444 links. We associated each of the 340 protein nodes from among the 652 nodes with a set of accession numbers, and then retrieved a set of biological events reported in MEDLINE on pairs of proteins corresponding to the accession number sets [10].

The set of retrieved pairs of proteins from MEDLINE can be used as basis for constructing a PPI network. Although recognition of protein pairs which appear in events contains errors, we can see significant differences of a PPI network to be constructed and a manually curated pathway.

**Figure 1:**
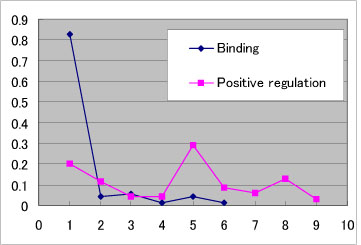
**The distribution of distances between the nodes.** The pair of nodes which is directly linked in the PPI network (all distances between the nodes are 1) seem to be associated through a sequences of links. The links in the PPI network corresponding to the different types of events show distinctly different distribution behaviours in terms of the length of corresponding paths in the pathway. The distance between nodes in the binding event tends to be close to 1, while the distance between nodes in the positive regulation tends to be distributed widely.

Table 1 shows how single proteins in the extracted pairs of proteins correspond to nodes in the manually constructed pathway. On the other hand, single links in the PPI network are expanded into paths in the pathway. That is, sequences of links appear to associate the pairs which are directly linked in the extracted pairs (all distances between nodes in a PPI network are 1). The links in the pairs corresponding to different types of events show distinctly different distribution behaviours in terms of the length of the corresponding paths in the pathway. Figure 1 shows the distribution of distances between nodes in the TLR pathway of the binding and positive regulation events.

**Table 1:** Distribution of one protein in the PPI to multiple nodes in the pathway.

Distribution	Frequency	Ratio
1	12,718	0.0737
2	16,180	0.0937
3	31,769	0.1841
4	49,408	0.2863
5	3,403	0.0197
6	18,205	0.1055
7	3,454	0.0200
8	6,435	0.0373
9	4,797	0.0278
10	2,082	0.0121
11	2,125	0.0123
12	35	0.0002
13	262	0.0015
18	46	0.0003
>20	21,655	0.1255

A single node in the PPI network tends to correspond to multiple nodes in the manually constructed pathway according to its state transitions. The distribution describes the number of nodes in the pathway that proteins in the pairs extracted from MEDLINE correspond to. The frequency, on the other hand, means how frequent each distribution is. That is, the frequency 16,180 of the distribution 2 means that protein names mapped to two nodes in the pathway occur 16,180 times in pairs extracted from MEDLINE. In the manually curated pathway a single protein in extracted pairs has 7.81 nodes on average which can be associated with it.

In order to see the reasons why such significant discrepancies appear between a PPI network and a pathway, we constructed a much smaller pathway, and carefully annotated a corpus based on the GENIA corpus.

### GENIA-pathway corpus and a NF-kB pathway

**Figure 2:**
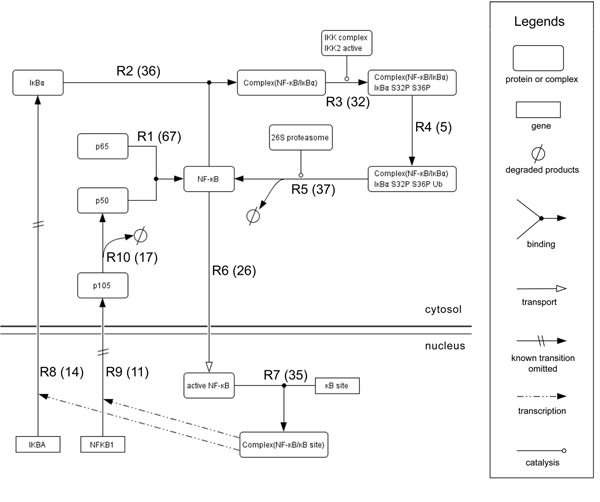
**NF-*k*B pathway GENIA version**. NF-kB pathway GENIA version is comprised of 16 entities, which are organized as 10 reactions. A brief explanation of the pathway is as follows: p65 binds to p50 to form a complex NF-kB (R1). NF-kB binds to IkBa in the cytosol (R2) that is rapidly phosphorylated on serine 32 and 36 (R3), ubiquitinated (R4), and degraded by 26S proteasome (R5). Freed NF-kB translocates to the nucleus (R6) and initiates the transcription of genes by binding to the kB site in their promoter region (R7). Because either IKBA or NFKB1 has a kB site in its promoter, NF-kB transcribes it to produce protein IkBa (R8) or p105 (R9), respectively. p105 is a precursor of p50 and constitutively processed to produce p50 (R10). The number of evidence sentence supporting the reaction is shown in parenthesis next to the ID of a reaction. P: phosphorylated, S: serine, Ub: ubiquitinated.

Among 1999 Medline abstracts in the GENIA corpus, 561 abstracts are indexed with the Mesh term, NF-kB. We call this subset the GENIA NF-kB pathway corpus, or the pathway corpus. Figure 2 shows a NF-kB pathway constructed manually based on the pathway corpus. It consists of 10 reactions in which 15 entities (proteins, genes, and their oligomers) participate. An ID is assigned to each reaction in the pathway with a set of evidence sentences for it. The number of evidence sentences is shown in the square bracket in Figure 2. Table 2 shows all of the evidence sentences for the reaction R6. Such an association between a reaction and its evidence sentences illustrates which sentences are judged by the specialist to be important for the construction of the portion of the pathway. Note that, as seen in Table 2, diverse expressions in text denote the same reaction, R6.

**Table 2:** Textual evidence for the reaction, R6 of the NF-kB pathway

PMID	SID	Textual Evidence from Literature
8430069	S4	**NF-kappa B**, which is rapidly translocated* from the cytoplasm to nucleus*
9095577	S5	translocation of** NF-kappa B*** into the nucleus*
8108414	S3	*nuclear* translocation of **NF-kappa B**
9032271	S4	**NF-kappaB **translocates* to the nucleus*
9442374	S5	**NF-kappa B **translocation* into the nucleus*
9804806	S8	*nuclear *translocation of** NF-kappa B**
2017258	S6	*nuclear* translocation of active **NF-kappa B**
8330898	S6	**NF-kappa B**, which is rapidly taken up* into nuclei*
8460169	S3	**NF-kappa B*** nuclear* localization
7520914	S7	*Nuclear *translocation of* cytosolic ***NF-kappa B**
7878466	S4	**NF-kappa B*** to translocate to the nucleus*
7925300	S4	*nuclear *translocation primarily of** p50-p65**
7929104	S5	translocation of **p65 and c-Rel NF.kappa B proteins ***from cytoplasmic stores to the nucleus*
9129050	S6	**NF-kappaB/Rel proteins **translocated* to nuclear fractions*
9224203	S6	**NF-kappa B **migrates* to the nucleus*
10197731	S3	translocation of the active **p65-50 heterodimer ***to the nucleus*
10202024	S8	**NF-kappaB **was translocated* to the nucleus*
10452760	S12	**NF-kappaB*** nuclear *translocation
7541794	S5	*nuclear* translocation of** the p50 and p65 subunits of the NF-kappa B transcription factor**
7594489	S1	*nuclear *translocation of** NF-kappa B (p50/p65)**
7594489	S8	*nuclear* translocation of** NF-kappa B p50/p65**
9344365	S11	**NFkappaB** to translocate* into the nucleus*
9442373	S2	**Transcription factor NF-kappa B** must be released from cytoplasmic inhibitory molecules (I kappa Bs) in order to move* to the nucleus*
7935451	S7	*nuclear *translocation of the active **NF-kappa B**
9366415	S10	**NF-kappa B ***nuclear *translocation
9804806	S5	release of **NF-kappaB/Rel proteins ***into the nucleus*

Textual evidence associated to the reaction R6 of the GENIA version NF-kB pathway. They are all extracted from the GENIA corpus and stored as GENIA pathway corpus. The PMID and SID (sentence ID) of the source of the textual evidence are given in the first and second column respectively. The reaction R6 represents localization of NF-kB from cytoplasm to nucleus. The text expressions referring to the NF-kB, the localization event and the location information are highlighted in bold, underlined, and italicized respectively.

**Figure 3:**
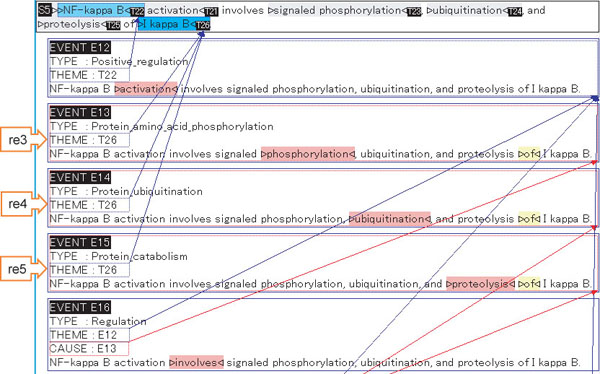
**Screenshot of one example from GENIA event annotation and corresponding reaction IDs.** One example from the GENIA pathway corpus is shown. Each sentence in the GENIA pathway corpus comes with event annotations and is mapped to the corresponding reactions in the NF-kB pathway. Event IDs E13, E14, E15 are the adopted events to the NF-kB pathway. Their mapped reaction IDs are also shown by the orange arrow boxes.

As a subset of the GENIA corpus, every sentence in the pathway corpus is accompanied by manually created event annotations. Figure 3 shows examples of event annotations, with the corresponding reactions in the pathway. This dual annotation of reactions in the pathway and events in text allows us to see not only how events in text are mapped to reactions in the pathway, but also what events or reactions implicit in text have to be inferred by bio-inferences.

### Context dependency of the mapping between text and a pathway

The first presented challenge is coping with the difficulty of identifying portions in a pathway relevant to expressions in text. The same biological entity (protein, molecule, etc.) may appear in more than one place in a pathway, since it is in different chemical states and thus possesses different properties. Unlike mapping proteins to their accession numbers in the traditional task setting [11], mapping named entities in text to nodes in a pathway is highly context dependent. Nodes in a pathway represent instances of a biomolecule, and single accession numbers are assigned to biomolecules. Table 3 depicts all of the entities in Figure 2. Among these entities, I4 and I5 are instances of the same biomolecule, the p50/p65, while I7, I8, and I9 are instances of the biomolecule, p50/p65/IkBa.

**Table 3:** Entities in the NF-kB pathway, the GENIA version

Id	Name	Biomolecule	State (attributes)
I1	p105	p105	
I2	p50	p50	
I3	p65	p65	
I4	NF-kB	p50/p65	Cytoplasm
I5	active NF-kB	p50/p65	Active, Nucleus
I6	IkBa	IkBa	
I7	Complex(NF-kB/IkBa)	p50/p65/IkBa	
I8	Complex(NF-kB/IkBa) IkBa S32P S36P	p50/p65/IkBa	Phosphorylated
I9	Complex(NF-kB/IkBa) IkBa S32P S36P Ub	p50/p65/IkBa	Phosphorylated, Ubiquitinated,
I10	kB site	kB site	
I11	Complex (NF-kB/kB site)	NF-kB/kB site	
I12	NFKB1	NFKB1	
I13	IKBA	IKBA	
I14	IKK complex	IKK complex	
I15	26S proteasome	26S proteasome	

The second column shows the name of each entity given by the biologist who created the pathway model. The biomolecules corresponding to each entity is given in the third column. The last column gives the state of each entity, which is represented as a set of predicates.

In text, an entity, that is, an instance of a biomolecule, is not represented as such. It is described by a reference to the corresponding bio-molecule (continuant) together with the surrounding textual context. One has to enumerate, from a given textual context, distinct biological states and then locate in these states the biological entities denoted by the biomolecule expression. Let us see the occurrences of NK-kB in the following two sentences:

*The active nuclear form of the NF-kappa B transcription factor complex is composed of two DNA binding subunits, NF-kappa B p65 and NF-kappa B p50, … (1493333-S2)*.

*Transcription factor NF-kappa B (p50/p65) is generally localized to the cytoplasm by its inhibitor I kappa B alpha (8319912-S2)*.

In the first sentence, the biological state in which NF-kB appears, is given by the preceding noun phrase “active nuclear form,” and we can thus associate the NF-kB reference with the entity I5 in Table 3. On the other hand, since the second sentence indicates that the NF-kB resides in the cytoplasm and interacts with IkBa, the NF-kB in this sentence has to be mapped to I7, which is an instance of the NF-kB/IkBa complex.

While these two sentences demonstrate that the same named entity expression ‘NF-kB’ must be mapped to different nodes in a pathway depending on the biological contexts in which they appear, the next example shows that an entity in one sentence must be mapped to different nodes in a pathway;

Stimulation of cells leads to a rapid phosphorylation of I Kappa B alpha, which is presumed to be important for the subsequent degradation. (7499266-S3)

**Figure 4:**
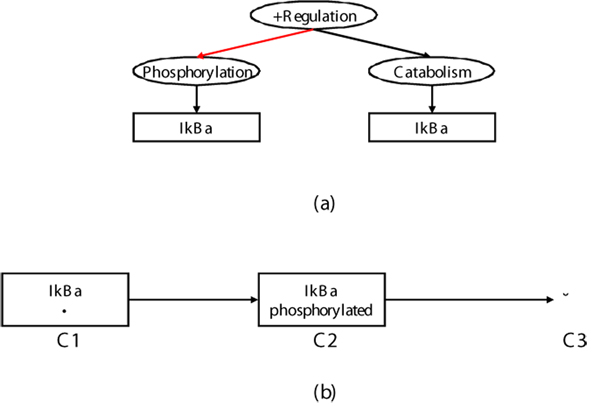
**Event representation in NL expression vs. pathway representation**. (a) Event annotation to the sentence “*Stimulation of cells leads to a rapid phosphorylation of I kappa B alpha, which is presumed to be important for the subsequent degradation*,” and (b) the pathway representation corresponding to it. In (a), the black and red arcs link an event with its theme and cause respectively. In (b), the black arcs link two entities representing change of the state.

This sentence makes references to two events: phosphorylation (R3), and degradation (R5), which occur in this order. Since a single event contrasts two biological states, the two events introduce three distinct states: before, between and after the two events. While IkBa appears only once in the sentence, it has three different states (un-phosphorylated, phosphorylated, and degraded product), and was thus mapped to three instances, which correspond to the nodes in a pathway. Figure 4 illustrates the difference between representations in text and a pathway for this sentence.

As exemplified here, for pathway-text association, we need to (1) recognize bio-molecule references from text (recognition of biomolecules), (2) capture biological contexts from the textual context (enumeration of biological contexts), and (3) find corresponding entities in a pathway (identification of instances in biological contexts). While step (1) is the classic Named Entity Recognition (NER) problem, steps (2) and (3) are newly introduced obstacles for the pathway-text association task.

### Integration of scattered information into a pathway

On the contrary, in some cases, entities in different texts may have to be mapped to the same node in a pathway. Table 2 shows all of the evidence sentences from the GENIA event corpus associated with the reaction R6. While at a glance, these evidence sentences appear to be different, they all refer to the same event: the translocation of NF-kB from the cytosol to the nucleus. In order to integrate information scattered over different articles into a pathway, these diverse surface expressions should be mapped to a single link (R6) in the pathway. All the typographic variants of NF-kappa B as well as its synonyms (p50-p60, etc., shown in bold), have to be recognized. Furthermore, the Localization event is described by using diverse predicates: *translocate*, *take up*, *migrate* and *move*, in diverse syntactic constructions (underlined). Simple information extraction techniques based on co-occurrences cannot deliver such fine-tuned recognition of events, and structure-based IE would be indispensable [6,12,13].

### New type of disambiguation problem

**Figure 5:**
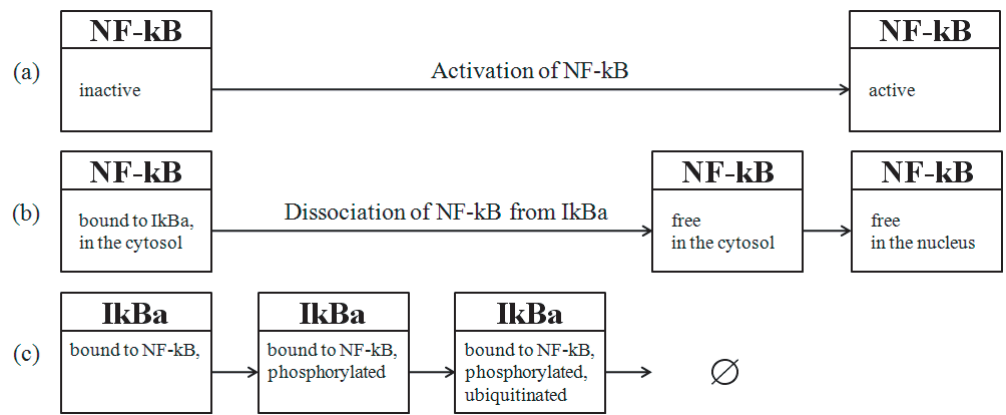
**NF-kB activation process from different perspectives**. A fragment (R3~R6) of the NF-kB lifecycle pathway in Figure 2 represents the process of NF-kB activation. (a), (b), and (c) illustrate a breakdown of the process from different perspectives: (a) from the perspective of the functional state of the NF-kB, (b) from the perspective of the molecular state of the NF-kB, and (c) from the perspective of the molecular state of the IkBa. Although (c) does not represent NF-kB directly, the state change of IkBa can be interpreted as a part of NF-kB activation process. It is reflected in the entities of the NF-kB/IkBa complex in the NF-kB pathway in Figure 1, which correspond to I7, I8 and I9 in Table 3.

Similarly, correspondence events reported in different papers and reactions in a pathway are not straightforward. Consider Figure 5, which shows a detailed interpretation of the reaction sequence, R3~R6. The main concern of the pathway is the state change of the NF-kB from the inactive to the active form (Figure 5(a)). The whole process is broken down into two sub-processes: the dissociation of NF-kB from IkBa, and the localization of NF-kB into the nucleus (Figure 5(b)). The dissociation process is described in more detail by the IkBa degradation process (Figure 5(c)). These three sequences constitute the whole process of NF-kB activation; however, no single sentence in the pathway corpus reveals this whole process. Each sentence focuses on any of three aspects: (a) the functional state of NF-kB, (b) the molecular state of NF-kB, or (c) the molecular state of IkBa. Let us examine the following two sentences:

n-Acetyl-L-cysteine (NAC), a potent antioxidant, blocked NF-kappa B activation caused by IL-4 and by anti-CD40 mAb. (8977531-S6)

The proteolytic degradation of the inhibitory protein MAD 3/I kappa B alpha in response to extracellular stimulation is a prerequisite step in the activation of the transcription factor NF-kappa B. (7565683-S2)

Since the first sentence focuses on external factors (NAC, IL-4 and anti-CE40 mAb) which cause the state change of NF-kB from inactive to active, NF-kB activation in this sentence refers the entire NF-kB activation process in Figure 5(a). In contrast, the second sentence discusses the process at the micro-level, and the NF-kB activation refers only to the localization portion in Figure 5(b), which is the moment at which the activation is accomplished. Thus, all aspects of information discussed in different articles must be integrated into a pathway. The pathway (Figure 2) is a result of such an effort by a biologist who has constructed the model pathway.

In Figure 5, the nodes in the same biological contexts are vertically aligned. This alignment assumes the basic knowledge of the biologist including: “the inactive NF-kB is equated with the NF-kB, which is bound to IkB in the cytoplasm,” and inferences such as, “the presence of the NF-kB which is bound to IkB in the cytoplasm also implies the presence of IkBa itself, which is bound to NF-kB in the cytoplasm” (symmetry). The three leftmost nodes aligned vertically in Figure 5 (the inactive NF-kB and the NF-kB and IkBa, which are bound to each other) are all equivalent and correspond to the entity I7 in Table 3.

### Constraints on sequences of multiple reactions

The second difficulty concerns the recognition of mutual relationships among multiple reactions. While the research on Information Extraction (IE) in the bio-medical domain has focused on the extraction of events or relations independently of the contexts in which they appear [14-16], a pathway construction needs to extract their mutual relationships in the form of causal chains [17-19]. Since exact precedence relationships among events are not usually expressed explicitly, we can only extract constraints on causal chains. At the integration stage, we construct a pathway which satisfies all constraints gathered from the whole set of articles.

Explicit causal expressions in text such as, “A causes B,” “A leads to B,” and “A activates B,” indicate constraints that “A precedes B”. Note that these expressions do not necessarily imply direct causation. It is common that there exist intervening reactions in the pathway between A and B in these expressions. Moreover, human biologists infer much richer constraints on sequences of reactions from expressions in text, other than explicit causal expressions. Consider the following sentence:

*A fraction of the phosphorylated form of I kappa B alpha remains physically associated with the NF-kappa B complex in vivo but is subject to rapid degradation, thereby promoting the nuclear translocation of the active NF-kappa B complex*.

There are four events mentioned in this sentence: (1) the binding of NF-kappa B complex and I kappa B alpha (R2), (2) the phosphorylation of I kappa B alpha (R3), (3) the degradation of I kappa B alpha (R5), and (4) the translocation of NF-kappa B complex (R6). More importantly, from this sentence, a biologist can extract relational constraints among these four events.

From the verb ‘remains’, s/he understands that the event in the subject (R3) does not distort the event in the objective complement (R2), thereby s/he may infer a constraint on the event sequence; that is, that R2 precedes R3. Another constraint is inferred by ‘is subject to.’ It indicates that a state after reaction (R3) in the subject is easily caught by another reaction (R5) in the object. Therefore, the constraint that R3 precedes R5 is inferred. Lastly ‘thereby’ shows that the event in the foregoing sentence (R5) is followed by the event in the subsequent sentence (R6). By putting them together, she/he successfully constructed the portion of these events in the pathway in Fig 2.

### Bio-inferences

The third difficulty comes from the use of biological domain knowledge. In general, at the stage of information integration in a pathway, biologists extensively use their background knowledge as well as the pathway at hand to infer pieces of information, which are implicit in text. We have to understand what cues in text trigger bio-inferences and what facts are to be inferred for pathway construction.

To elucidate the inference process, we classified the evidence sentences according to whether they contain direct expressions of the identified reactions or not (Table 4). Evidence sentences are classified as direct when they contain annotated events in the GENIA event annotation. The events in the GENIA corpus were independently annotated by a group of annotators. The sentences judged as indirect do not contain such corresponding annotated events but nonetheless were identified as evidence sentences. Table 4 shows that evidence sentences were distributed evenly between direct and indirect ones. In the following, we identify what constitutes indirect cues and discuss what types of bio-inferences are triggered by them. The analysis reveals that highly domain-dependent cues trigger bio-inferences.

**Table 4:** The dichotomy of the sentence expression for pathway representation: direct or indirect.

**reaction ID**	**reaction type**	**expected annotation class**	**direct (annotated)**	**indirect (not annotated)**
**R1**	Binding	Binding	11	56
**R2**	Binding	Binding	16	20
**R3**	phosphorylation	Protein_amino_acid_phosphorylation	19	1
	+ site information	Protein_amino_acid_phosphorylation	2	5
	+ modifier information	+ (any) Regulation	4	1
**R4**	ubiquitination	Protein_ubiquitination	5	0
**R5**	degradation	Protein_catabolism	27	1
	+ modifier information	+ (any) Regulation	6	3
**R6**	translocation	Localization	26	0
**R7**	Binding	Binding	23	12
**R8**	gene expression	Gene_expression (or Transcription + Translation)	5	9
**R9**	gene expression	Gene expression (or Transcription + Translation)	1	10
**R10**	processing	Protein_processing	5	12
		total	150	130

The sentences for pathway representation of each reaction were distributed by a direct or indirect expression. The direct expressions were annotated by ‘expected annotation class’ using GENIA event annotation, while the indirect expressions were not. The latter needs some ‘inference’ by the biological domain knowledge to construct the corresponding pathway.

### Classification of bio-inference for pathway construction from text

We analysed evidence sentences regarded as indirect to see whether specific textual cues exist to trigger the inference, and classified them according to their relationships with the inferred reactions. The analysis resulted in six major inference schemas shown in Table 5. An example sentence of each scheme is shown in Table 6.

**Table 5:** The breakdown of the indirect expressions by ‘inference’ scheme

**reaction ID**	**reaction type**	n	1		2		3		4	5	6

			a	b	a	b	a	b			
R1	Binding	56		56							
R2	Binding	20			3	15				2	
R3	phosphorylation	1		1							
	+ site information	5					4	1			
	+ modifier information	1							1		
R5	degradation	1		1							
	+ modifier information	3						1			2
R7	Binding	12		3	2	6		1			
R8	gene expression	9	3		4				2		
R9	gene expression	10	3	2	4				1		
R10	processing	12	11	1							

The indirect expressions were subdivided by ‘inference’ scheme. Biologists often build a pathway by ‘inference’ with their biological domain knowledge from indirect expressions in the text. There are nine inference schemes when regarding indirect expressions, which indicate specific reactions. The figures are the number of sentences in each ‘inference’ scheme. Class 1a: state(s) of entity(-ies) before reaction, 1b: state(s) of entity(-ies) after reaction, 2a: function(s) of entity(-ies) before reaction, 2b: function(s) of entity(-ies) after reaction, 3a: influence of state change(s) of entity(-ies), 3b: influence of functional change(s) of entity(-ies), 4: related reaction, 5: reverse reaction, 6:characteristic of reaction.

#### 1a/b. State(s) of entity(-ies) before or after reaction

From the existence of a certain entity (cue) the biologist inferred the associated reaction. Example cues of this class are **precursor** (1a, ex1) and **heterodimer** (1b, ex2) (shown in boldface in Table 6). In general, biologists know that a precursor should be processed to the mature protein and that a heterodimer represents two distinct proteins coming together. These cues trigger inference to assume a processing and binding event, respectively.

**Table 6:** The example sentences of each ‘bio-inference’ scheme

class		example		inferred reaction IDs
1	a	ex1	p50 is translated as a **precursor** of 105 kDa.	R10
	b	ex2	CD23-induced NF-kappaB is a **heterodimer** composed of p65/p50 subunits.	R1
2	a	ex3	RelA contains a **high-affinity binding site** for its cytoplasmic inhibitor, I kappa B alpha.	R2
	b	ex4	I kappa B-alpha inhibits transcription factor NF-kappa B by **retaining it in the cytoplasm**.	R2
3	a	ex5	When either **serine-**32 or **serine-**36 of I kappa B-alpha was **mutated**, the protein did not undergo signal-induced phosphorylation.	R3
	b	ex6	Pretreatment of the cells with the **proteasome inhibitor** N-Ac-Leu-Leu-norleucinal inhibits this ligand-induced degradation of the human I kappa B alpha protein.	R5
4		ex7	**As observed with** I kappa B alpha, nuclear RelA stimulates p100 mRNA and protein expression.	R8
5		ex8	Nuclear expression of NF-kappa B occurs after its induced **dissociation** from its cytoplasmic inhibitor I kappa B alpha.	R2
6		ex9	A failure to degrade IkappaB-alpha pretreated with PAO is not due to its inhibitory effect on **proteasomal** degradation.	R5

The example sentences of each class of ‘bio-inference’ scheme are shown with their inferred reaction IDs. The linguistic cues to infer some reaction are shown in boldface.

#### 2a/b. Function (s) of entity(-ies) before or after reaction

The next class is the inference scheme based on the existence of a certain entity(-ies) with a specific function. The example cues of these classes are the **high-affinity binding site** (ex3) and **retaining it in the cytoplasm** (ex4), respectively. These indicate that a protein mentioned in the sentence has a specific function. The protein that has a binding function with some protein with high affinity will bind it, ten to one, and the one that has a function of retaining another protein never displays its ability without binding to it. The evidence sentences of these two cues appear for R2.

#### 3a/b. Influence of state or functional change(s) of entity(-ies)

This is the class of inferring a latent reaction from the influence caused by state or functional change. Examples of cue phrases of this type are ‘**serine, mutated**’ (3a, ex5), and ‘**proteasome inhibitor**’ (3b, ex6), respectively. Due to the state change of the serine mutation, the protein was not phosphorylated. On the flipside, biologists recognize the reaction that the protein is to be phosphorylated on serine if not mutated (R3). Treatment with a proteasome inhibitor changes the function of the proteasome, thereby inhibiting the degradation event of I kappa B alpha (R5). Biologists also know I kappa B alpha is to be degraded by proteasome if not inhibited. Overall, this class of bio-inference is an association of the reaction normally occurring if something (state or function) is not changed.

#### 4. Related reaction

This class of cues is based on the strong association between two reactions. It infers a reaction unmentioned in text from an explicit reaction. The example of the linguistic cue phrase of this class is ‘**As observed with**’ (Table 4, ex7). The example does not show that any reaction occurred to I kappa B alpha directly. However, because of the expression: ‘*As observed with*,’ and the biological knowledge that RelA is a transcription factor, and that I kappa B alpha is a protein produced from its gene through the transcriptional and translational event by a transcription factor, biologists recognize the existence of the reaction that RelA transcribes the mRNA of IkBa gene, and IkBa protein will be induced. Further, the gene expression event occurred to IkBa by RelA (R8). Thus, the linguistic cue of this class appears in the sentence structure.

#### 5. Reverse reaction

This class is the inference from the reverse reaction. The example, ex8 describes the dissociation event that occurred between ‘NF-kappa B’ and ‘I kappa B alpha.’ The dissociation must be preceded by binding event (R2), because all proteins are produced as a single molecule. However, the reverse reaction does not always occur; whether the reverse reaction exists or not depends on the domain knowledge.

#### 6. Characteristic of reaction

The last class is the inference scheme based on a qualifier to show a characteristic of a reaction. The example, ex9 shows the degradation event that occurred to ‘IkappaB-alpha’ directly (R5), but does not indicate what caused the reaction, or how it was reaction. Biologists will pay attention to the cue qualifier ‘**proteosomal.**’ They know that, as biological domain knowledge, its substantive form ‘proteasome’ is a name of a huge protein complex that causes protein degradation. Thus, they can infer that the modifier should be the proteasome of the degradation event on IkBa from the qualifier ‘proteasomal’ that shows the characteristic of a reaction.

## Discussion

Due to the complexity and size of the pathways being, and to be constructed, TM tools for facilitating their construction and maintenance become crucial for bio-medical research. At the same time, text retrieval based on pathways will become an effective means by which biologists may gain access to articles relevant to their interests. For all such scenarios for TM tools to be materialized, technical challenges involved in associating text with pathways have to be properly understood and formulated.

In this paper, we identified the three challenges. The first one, identification of the mapping position of a specific entity in a pathway, is beyond the traditional challenge tasks of accession number assignment for proteins [11] or protein-protein interactions (PPI) [16]. The resulting comparison between a PPI network and a pathway shows that the same biomolecules appear in several places in a pathway, thus making it necessary for us to associate bio-molecular expressions in text with one of their occurrences in a pathway. The correspondence is highly context dependent.

The second challenge is the recognition of constraints on sequences of multiple reactions. While temporal sequences of events have been studied in the general domain, their techniques are mostly concerned with the treatment of temporal expressions [17]. On the other hand, as exemplified in this paper, we have to recognize causal relationships among events, which require inferences based on the domain knowledge as well as subtle textual cues in text, instead of explicit temporal expressions.

The third and the most difficult of the challenges is the problem involved in formulating and implementing bio-inferences. Half of all crucial evidence sentences do not contain an explicit description of events. The specialist inferred implicit events, and the inferences seem to be triggered by specific fragments of expressions in text. While logical or deductive inferences based on the domain knowledge certainly play a role in the process [20], the inferences here are more akin to associative and plausible inferences [21]. How to formulate and exploit such inferences in TM would be the greatest challenge of all.

**Figure 6:**
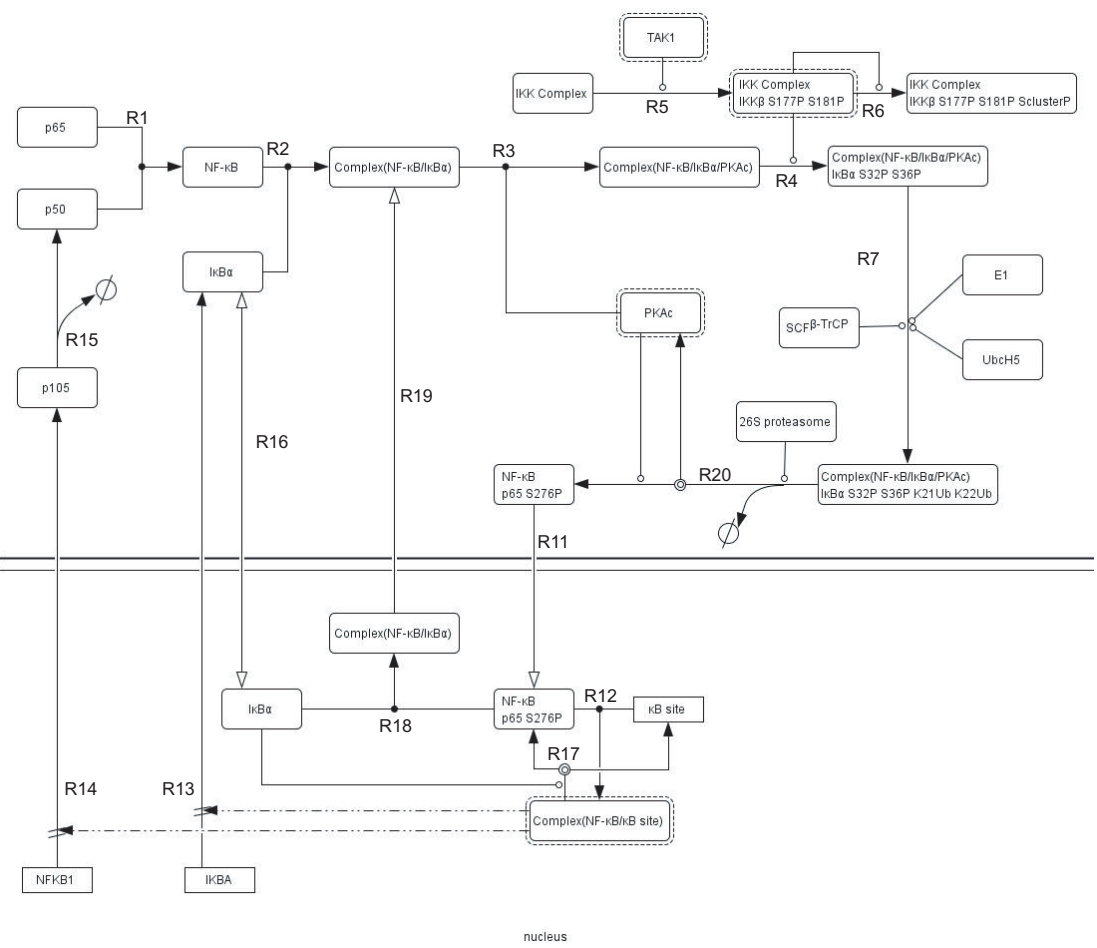
**NF-kB pathway full-text version**. The NF-kB pathway full-text version, bigger than GENIA version, has 28 entities and 17 reactions. The additional reactions are as follows: R11: binding between PKAc and Complex(NF-kB/IkBa), R12: phosphorylation of IKK Complex by TAK1, R13:phosphorylation of IKKb Serine cluster, R14: degradation of IkBa by 26S proteasome + dissociation of PKAc from NF-kB + NF-kB phosphorylation by PKAc occur almost at the same time, R15: shattling of IkBa between cytosol and nucleus, R16: dissociation of Complex(NF-kB/kB site) by IkBa, R17: binding between NF-kB and IkBa in the nucleus, R18: translocation of Complex(NF-kB/IkBa) from nucleus to the cytosol.

The analysis given in this paper has been made on the pathway corpus, which has been built based on the GENIA corpus. Since the GENIA pathway corpus is a highly biased corpus [22], and the size is currently not large enough, we are now working on scaling up the pathway corpus by using full papers, and extending the corpus beyond the GENIA set. Figure 6 shows a fuller version of the NF-kB pathway being constructed by a collection of full papers from the whole Medline. To enlarge the pathway corpus as a rich resource, we will append annotations to the collected relevant sentences depending on whether the text is relevant, which reaction it indicates, whether the expression is ‘direct’ or ‘indirect,’ and its inference scheme in cases where the expression ‘indirect.’ The stored data will be also annotated by the GENIA method of event annotation. In the future we will be engaged in another kind of pathway such as epidermal growth factor receptor signalling pathway thus establishing a consistent method of the pathway annotation for the automatic building of a pathway from text.

## Conclusions

In this study, we presented two new resources: pathway corpus and its corresponding NF-kB pathway, whose mapping among pathway and text is compared with annotated events. Based on a detailed corpus study, we formulated three challenges for TM tools. We also revealed that inferences triggered by highly domain-dependent cues play a central role in recovering events implicit in text but crucial for text-pathway association. We believe the corpus-based study presented in this paper is an important first step for addressing the new TM technology for pathway construction.

## Methods

### Construction of a PPI network and its mapping to a pathway

The PPI network for the proteins in the TLR pathway is constructed by using an IE system [23]. The IE system uses a full parser [24] to reveal the semantic structures of sentences, and then applies simple rules to identify event types. The event type recognition is based on linguistic clues developed by our previous work [8]. A dictionary-based protein name recognizer is used to map the protein names to a set of accession numbers [10].

The distribution of path lengths for the binding and positive regulation events (Figure 1) are calculated after KO identifies the corresponding nodes in the pathway for each protein in the sentences in which the event is recognized. Moreover, the distribution of path lengths for pairs in all extracted events is calculated by assuming that the two nodes which give the shortest path are the nodes corresponding to the two proteins of a given pair. Therefore, the lengths tend to be estimated to be shorter than in actuality.

### Event annotation and the pathway corpus

GENIA event annotation was made on half of the GENEA corpus [8,25], and consists of 1,000 Medline abstracts. It contains 9,372 sentences in which 36,114 events are identified. The annotation was carried out over the process of two years by a group of annotators (four to six biologists) and one coordinator (co-author of the paper, TO). In order to avoid inter-annotator discrepancy, the instructions were given to prevent them from making free inferences.

On the contrary, the pathway corpus was constructed by a single biologist (co-author of the paper, KO) who has a substantial amount of experience in constructing pathways from literature [9,26]. KO read all of 5, 223 sentences in the 561 abstracts and constructed the pathway, all the links of which were associated with a set of evidence sentences taken from 5,223 sentences. Unlike event annotation, KO established those links freely and then analyzed, retrospectively, what inferences were made in the process.

## Competing interests

The authors declare that they have no competing interests.

## Authors' contributions

KO carried out the construction of the pathway corpus, NF-kappa B pathway, manually annotated to link to the pathway, analyzed the inference scheme, and drafted the manuscript. JDK participated in the design of the study, carried out an investigation about the mapping position in a pathway, and drafted the manuscript. TO chiefly carried out the GENA event annotation and drafted the manuscript. DO and TM constructed a PPI network and mapped it to a Pathway, and drafted the manuscript. YT participated in the design of the study and drafted the manuscript. JT conceived of the study, participated in its design and coordination, and helped to draft the manuscript. All authors read and approved the final manuscript.
